# Upregulation of PD-1/PD-L1 and downregulation of immune signaling pathways lead to more severe visceral leishmaniasis in undernutrition mice

**DOI:** 10.1186/s13071-023-06018-2

**Published:** 2024-01-08

**Authors:** Jinlei He, Jianhui Zhang, Xuechun Liao, Yuying Xiao, Jiao Li, Zhiwan Zheng, Dali Chen, Jianping Chen

**Affiliations:** Department of Pathogenic Biology, West China School of Basic Medical Sciences and Forensic Medicine, Sichuan University, Chengdu, China

**Keywords:** Leishmaniasis, *PD-1*, *PD-L1*, Undernutrition, RNA-seq

## Abstract

**Background:**

Leishmaniasis is mainly prevalent in tropical and subtropical developing countries, where chronic undernutrition often co-exists. Undernutrition is reported to promote the progression of leishmaniasis, but its immune mechanisms have not been fully elucidated.

**Methods:**

To simulate chronic undernutrition of patients in epidemic areas and explore the immune mechanism of undernutrition promoting leishmaniasis, BALB/c mouse models with different nutritional imbalances were established, including undernutrition 75%, undernutrition 65% and obesity mouse models. After infection with *Leishmania donovani* in these model mice, we focused on evaluating the progress of leishmaniasis in the spleen and liver, the expression of important immunosuppressive and immunoactivation molecules, and changes of spleen transcriptome. The immune signaling pathways enriched by differentially expressed genes and hub genes were analyzed.

**Results:**

The results showed that among the mouse infection models, undernutrition 75% + infection group had the highest parasite load in the spleen and liver at the 8th week post-infection, possibly due to the continuous increase of *PD-1*, *PD-L1* and *TCR*. Spleen RNA-seq results suggested that some immune signaling pathways were downregulated in undernutrition 75% + infection group, including neutrophil extracellular trap formation, IL-17 signaling pathway, natural killer cell-mediated cytotoxicity, etc. Among them, neutrophil extracellular trap formation pathway had the largest number of downregulated genes. This also explained why undernutrition 75% + infection group had the highest parasite load. Through PPI network analysis, hub genes such as *Lcn2*, *Ltf*, *Mpo*, *Dnaja1*, *Hspa1a*, *Hspa1b* and *Hsph1* were screened out and might play important roles in the process of undernutrition promoting leishmaniasis.

**Conclusions:**

Undernutrition upregulated *PD-1* and *PD-L1* expression and downregulated immune signaling pathways in mice with visceral leishmaniasis. The signaling pathways and hub genes may serve as drug targets or intervention targets for the treatment of leishmaniasis patients with undernutrition.

**Graphical Abstract:**

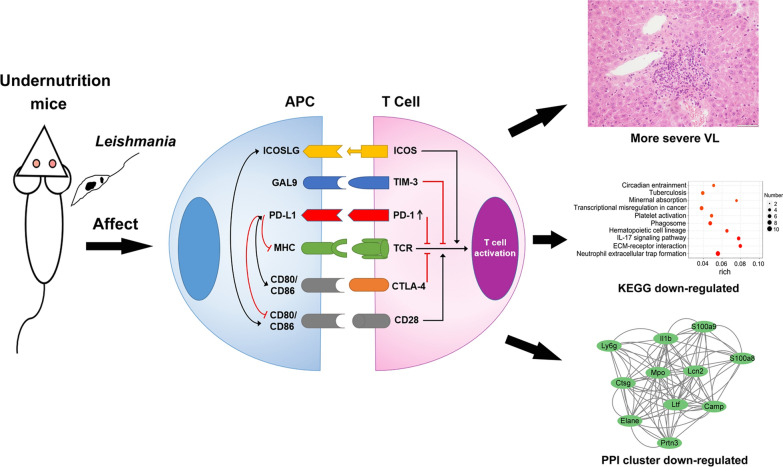

**Supplementary Information:**

The online version contains supplementary material available at 10.1186/s13071-023-06018-2.

## Background

Leishmaniasis is one of the most devastating neglected tropical diseases in the world. Currently, the disease is mainly prevalent in tropical and subtropical developing countries, covering about 90 countries [1]. The World Health Organization (WHO) reported that more than 1 billion people live in leishmaniasis-endemic areas and are at risk of infection. Visceral leishmaniasis (VL) is the deadliest form of leishmaniasis, which can lead to pathological changes in liver, spleen, bone marrow and lymph nodes. VL mainly affects developing countries, where chronic undernutrition often co-exists. Undernutrition is an important risk factor of leishmaniasis [2]. A study by Zacarias et al. demonstrated that the parasite burden of *Leishmania infantum* in children < 5 years old with acute moderate to severe malnourishment was almost three times greater than that of better-nourished children [3]. Harhay et al. studied 29,570 consecutive VL patients from 20 locations in Brazil, East Africa, Nepal and India between 1997 and 2009 and found that the proportion of malnourished VL patients < 5 years old was high in these areas, even as high as 39.3% in the Upper Nile region in Southern Sudan [4]. Moreover, studies have suggested that undernutrition may speed the progression of leishmaniasis, and leishmaniasis in turn can also aggravate undernutrition [5, 6].

The mechanisms of undernutrition aggravating leishmaniasis are related to immune damage and immunosuppression [7, 8], but the mechanistic relationship between undernutrition and the course of VL at the molecular and cellular levels is not fully understood. A better understanding of these mechanisms might offer new opportunities for prevention or therapeutic dietary intervention [9]. As important immunosuppressive molecules, programmed cell death protein 1 (PD-1) and its ligand programmed cell death ligand 1 (PD-L1) can promote the exhaustion of immune cells [10] and play important roles in the progression of leishmaniasis. It was found that both CD4^+^ and CD8^+^ T cells were exhausted in BALB/c mice 56 days after *Leishmania amazonensis* infection, with decreased T cell proliferation and increased PD-1 and PD-L1 expression [11]. After blocking PD-1/PD-L1 signaling pathway, both CD4^+^ and CD8^+^ T cell exhaustions were partially restored, and the spleen parasite load was reduced [11, 12]. Blocking PD-1/PD-L1 pathway has received extensive attention in the study of immunotherapy [13] and may become a new direction for the treatment of leishmaniasis. However, the expressions of PD-1 and PD-L1 and related molecular mechanisms in malnourished VL patients are not clear.

To investigate changes of immune response against *Leishmania* parasites during undernutrition, we established different nutritional imbalance models in BALB/c mice and infected these model mice with *Leishmania donovani*. At the 5th and 8th weeks after infection, liver and spleen weight, liver and spleen parasite loads, liver pathological changes, spleen lymphocyte subset proportions, serum antibody titers and serum cytokines were detected in each group to evaluate the progression of VL. The expression of T cell exhaustion, T cell activation, apoptosis and Toll-like receptor-related genes was measured by quantitative PCR (qPCR). The proportions of spleen T-lymphocyte subsets and their expression of PD-1 were detected by flow cytometry. Moreover, the transcriptome of spleen tissues from infected model mice was sequenced to explore the possible molecular mechanisms of undernutrition aggravating leishmaniasis and to screen important immune signaling pathways and hub genes involved.

## Methods

### Mouse models

The animal experiment was approved by the Sichuan University Medical Ethics Committee (approval no. K2022014). Eighty female BALB/c mice aged 6–8 weeks were provided by the Laboratory Animal Center of Sichuan University and co-housed in standard conditions with four mice per cage. The chronic nutritional imbalance model mice were divided into three groups (*n* = 20/group). Group one was fed a maintenance diet with 75% daily intake of normal mice (undernutrition 75% group), group two was fed 65% daily intake of normal mice (undernutrition 65% group) [14, 15], and group three was fed a high-fat and high-sugar diet containing about 15% fat and 25% sugar (obesity group). Normal control mice (*n* = 20/group) were fed a maintenance diet containing ≥ 4% fat and no added sugar. The general condition and activity of mice were observed daily, and the weight of mice was monitored once a week.

### *Leishmania* infection

*Leishmania donovani* strain MHOM/CN/90/9044 is preserved by our laboratory [16, 17]. The promastigotes of *Leishmania* were cultured in vitro in M199 medium (Hyclone, Thermo Fisher) containing 10% fetal bovine serum (PAN-Biotech, Germany) and 1% penicillin–streptomycin (Hyclone, Thermo Fisher). The working concentration for penicillin was 100 U/ml, and for streptomycin was 100 μg/ml. When the promastigotes grew to the stationary phase, they were intraperitoneally inoculated into nutritional imbalance model mice and normal mice with 3 × 10^7^ promastigotes per mouse at the 5th week after modeling. The nutritional imbalance model mice continued to be fed according to the previous modeling protocol. After infection, experimental mice were divided into eight groups: normal group (*n* = 12), obesity group (*n* = 12), undernutrition 75% group (*n* = 12), undernutrition 65% group (*n* = 12), normal + infection group (*n* = 8), obesity + infection group (*n* = 8), undernutrition 75% + infection group (*n* = 8) and undernutrition 65% + infection group (*n* = 8). In four uninfected groups, there were 12 mice in each group, and four of them were used to evaluate the success of undernutrition and obesity mouse models.

### Parasite load and liver pathology

Liver and spleen tissue samples were obtained from mice in each group at the 5th and 8th weeks after infection. The spleen and liver tissues of mice were weighed, and RNA from spleen and liver tissues was extracted using the RNAeasy Animal RNA Isolation Kit (Beyotime, China) and reverse transcribed into cDNA using the BeyoRT II First Strand cDNA Synthesis Kit (Beyotime, China). The contents of *Leishmania* 18S ribosomal RNA (primers were shown in Table 1) in spleen and liver tissues were detected by qPCR with SYBR green I (Beyotime, China) as a fluorescent dye to evaluate parasite load after infection. The remaining liver tissues were fixed to make pathological sections and stained with hematoxylin-eosin to assess the pathological changes.

**Table 1:** Primers used in this study

Gene	Sequence (5′–3′)	Amplified gene size (bp)	GeneBank ID	Amplification temperature (℃)
*18S*	F: ACCGTTTCGGCTTTTGTTGG	196	XR_002966730.1	59
	R: CCCCGAACTACCCTCCTTCA			
*Bax*	F: AATTGGAGATGAACTGGACAGCA	153	NM_007527.3	56
	R: AAGTAGAAGAGGGCAACCACGCG			
*Bcl-2*	F: ACTTCTCTCGTCGCTACCGT	136	AH001858.2	56
	R: ACAATCCTCCCCCAGTTCAC			
*Caspase-3*	F: GCAGCTTTGTGTGTGTGATTC	136	BC038825.1	56
	R: AGTTTCGGCTTTCCAGTCAG			
*CD28*	F: CCCCTGCTTGTGGTAGA	177	NM_007642.4	56
	R: GTTGAACTCGGCATTCG			
*CTLA-4*	F: GTGGGCTTCCTAGATTACC	140	NM_009843.4	56
	R: ACAAAGTATGGCGGTGG			
*GAPDH*	F: TCTTGGGCTACACTGAGGAC	126	GU214026.1	56
	R: CATACCAGGAAATGAGCTTGA			
*ICOS*	F: CACTGCTTCAGGGCTTAG	133	NM_017480.2	56
	R: GGGTGGGAGGGAATGTA			
*PD-1*	F: TGTGCCTGGAAATGGAG	110	NM_008798.3	56
	R: GGTGGCTTTAGGTGCTG			
*PD-L1*	F: GCCTCAGCACAGCAACTT	157	BC066841.1	56
	R: CGTGATTCGCTTGTAGTCC			
*TCR*	F: GCTGGTGGGTGAATGG	146	AH002089.2	56
	R: GCAGCGGAAGTGGTTT			
*Tim-3*	F: CACCATCGAGGAGAACG	139	NM_134250.2	56
	R: AAGGGAAATACCAAAGTCAG			
*TLR2*	F: TGCCACCATTTCCACG	184	AF185284.1	56
	R: AGGGCGGGTCAGAGTT			
*TLR4*	F: AGCATGGACCTTACCGG	127	NM_021297.3	56
	R: TAGCCTCTTCTCCTTCAGATT			
*TLR9*	F: TCTCCAACCGTATCCACC	138	AF348140.1	56
	R: TGGGCTCAATGGTCATG			

*18S*: 18S ribosomal RNA; *Bax*: BCL2-associated X protein; *Bcl-2*: B cell lymphoma-2; *Caspase-3*: cysteinyl aspartate-specific proteinase-3; *CD28*: cluster differentiation 28; *CTLA-4*: cytotoxic T lymphocyte-associated antigen-4; *GAPDH*: glyceraldehyde-3-phosphate dehydrogenase; *ICOS*: inducible costimulator; *PD-1*: programmed cell death protein 1; *PD-L1*: programmed cell death 1 ligand 1; *TCR*: T cell receptor; *Tim-3*: lymphocyte activation gene-3; *TLR2*: Toll-like receptor 2; *TLR4*: Toll-like receptor 4; *TLR9*: Toll-like receptor 9. The amplification curve for the qPCR results is shown in Additional file 1: Fig. S1

### qPCR to detect the expression of immune genes in spleen and liver

The steps of RNA extraction from spleen and liver tissues and reverse transcription into cDNA were the same as described above. The expression of T cell exhaustion genes *CTLA-4*, *PD-1*, *PD-L1*, *Tim-3*, T cell activation genes *CD28*, *ICOS*, *TCR*, apoptosis genes *Bax*, *Bcl-2*, *Caspase-3* and Toll-like receptor genes *TLR2*, *TLR4*, *TLR9* was detected by qPCR. Using normal uninfected mice samples as controls and *GAPDH* as a reference gene, the relative expression of these genes was calculated by the 2^(−∆∆Ct)^ method. All the primers are shown in Table 1.

### Flow cytometry detection of spleen T lymphocyte subsets and PD-1 expression

The spleens of mice in each group were made into cell suspensions at the 5th and 8th weeks after infection. The cell concentration was adjusted to 100,000 cells per milliliter using PBS. For each sample, 100 μl cell suspension was transferred into a flow tube with 1 μg FITC Hamster Anti-Mouse CD3e, PerCP-Cy5.5 Rat Anti-Mouse CD4, PE-Cy7 Rat Anti-Mouse CD8a and PE Hamster Anti-Mouse CD279 (PD-1) (BD, America) added, respectively. T lymphocytes were detected using CytoFLEX (Beckman) flow cytometry, and data were analyzed by FlowJo software (https://www.flowjo.com/). The CD3-positive cell population was selected for gating, and the proportions of CD3^+^CD4^+^, CD3^+^CD8^+^, CD3^+^CD279^+^, CD3^+^CD4^+^CD279^+^ and CD3^+^CD8^+^CD279^+^ positive cells were analyzed.

### Serum anti-*Leishmania* antibody titers

Mouse serum samples from each group were collected at the 5th and 8th weeks after infection. The serum total immunoglobulin G (IgG), IgG1 and IgG2a antibody titers were detected by indirect enzyme-linked immunosorbent assay (ELISA). The promastigotes of *Leishmania* were freeze-thawed five times in liquid nitrogen and centrifuged to obtain the supernatant as soluble *Leishmania* antigens (SLAs). The protein concentration of SLAs was detected using the BCA Protein Assay Kit (Beyotime, China), and SLAs were coated in 96-well plates with 100 μl per well at a concentration of 2 μg/ml. Serum samples from each group were diluted in 0.1% PBST using the serial dilution method to determine the antibody titers. Correspondingly, diluted sera from normal mice were used as negative controls. The secondary antibodies were HRP-conjugated affinipure goat anti-mouse IgG (H + L), HRP-conjugated affinipure goat anti-mouse IgG Fc γ subclass 1 specific and HRP-conjugated affinipure goat anti-mouse IgG Fc γ subclass 2a specific (Proteintech, America), respectively.

### Serum lipids, transaminases and cytokines

The contents of total cholesterol (TC), triglyceride (TG), high-density lipoprotein cholesterol (HDL-C), low-density lipoprotein cholesterol (LDL-C), alanine aminotransferase (ALT) and aspartate aminotransferase (AST) in mouse serum were detected using kits from Nanjing Jiancheng Bioengineering Institute. The serum interferon gamma (IFN-γ), interleukin 4 (IL-4), interleukin 10 (IL-10), interleukin 12 p70 (IL-12 p70) and tumor necrosis factor alpha (TNF-α) were detected using the OptEIA Mouse IFN-γ ELISA Set, OptEIA Mouse IL-4 ELISA Set, OptEIA Mouse IL-10 ELISA Set, OptEIA Mouse IL-12 (p70) ELISA Set and OptEIA Mouse TNF-α ELISA Set (BD Biosciences, USA).

### Transcriptome sequencing analysis of mouse spleen

After 8 weeks of infection, spleen tissues from normal mice, normal + infection mice, undernutrition 75% + infection mice and obesity + infection mice were sent to Shanghai Personalbio Technology Co., Ltd., for transcriptome sequencing. After RNA extraction, purification and library construction, next-generation sequencing technology was used for paired-end sequencing on these libraries based on the Illumina sequencing platform. The filtered high-quality sequences were compared to the reference genome Mus_musculus.GRCm39.dna.toplevel.fa. According to the comparison results, the expression level of each gene was calculated, and the samples were analyzed for expression quantity, expression differences, functional enrichment of differentially expressed genes (DEGs) and protein network interaction.

HTSeq [18] was used to compare the read count value of each gene as the original expression amount, and FPKM (Fragments Per Kilo bases per Million fragments) [19] was used to normalize the expression amount. Using DESeq for differential analysis of gene expression, the conditions for screening DEGs were: expression fold |log2FoldChange|> 1, significant *P*-value < 0.05 [20]. The R language Pheatmap package [21] was used to perform bidirectional clustering analysis on the union of differential genes and samples of all comparison groups. Kyoto Encyclopedia of Genes and Genomes (KEGG) [22] and Gene Set Enrichment Analysis [23] were performed to identify signaling pathways with the most significant DEG enrichment. Finally, the Search Tool for the Retrieval of Interacting Genes (STRING) [24] and Cytoscape 3.9.1 software [25] were used for protein-protein interaction (PPI) network analysis and hub gene cluster prediction. The interactions among DEGs were selected with a confidence score of > 0.4 [26], and the signaling pathways enriched by hub genes were predicted to reveal the role of these genes. The important hub genes were inputted into the UniProt database [27] to analyze their functions.

### Statistical analysis

Statistical analysis and graphing were performed using GraphPad Prism 8 software. Data were analyzed using one-way ANOVA and expressed as the means ± standard deviations. Kruskal-Wallis test was used when the samples did not follow a Gaussian distribution. Significant differences were determined and designated with asterisks as follows: *P < 0.05, **P < 0.01, ***P < 0.001.

## Results

### Body weight, spleen and liver weight, liver granulomas and parasite load

**Fig. 1:**
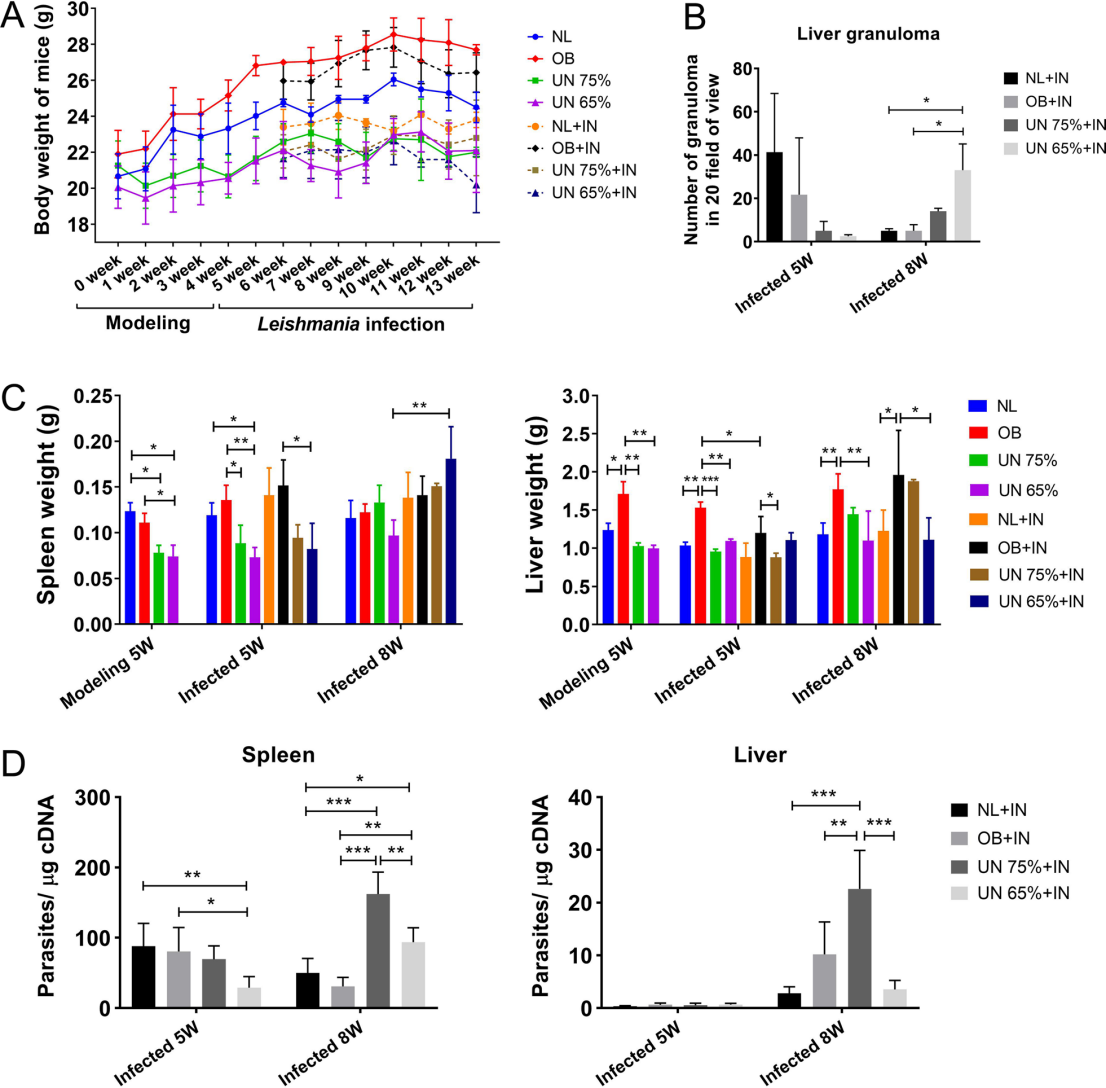
Body weight, spleen and liver weight, liver granulomas and parasite loads of spleen and liver in mice. IN: infection; NL: normal mice; OB: obesity mice; UN: undernutrition mice. **A** Body weight changes of different model mice. Undernutrition and obesity mouse models were established by feeding mice with different diets at 0–5 weeks, and each group of mice was infected with *Leishmania* parasites at the 5th week after modeling. **B** Liver granuloma count of mice in each group at the 5th and 8th weeks post-infection. **C** The weight of spleen and liver in mice at the 5th week after undernutrition and obesity modeling and at the 5th and 8th weeks post-infection. **D** Parasite loads of spleen and liver at the 5th and 8th weeks post-infection

The body, spleen and liver weights of mice are shown in Fig. 1A, C. The body weight of obesity mice was the heaviest, which was significantly higher than that of normal (*P* = 0.0007) and undernutrition mice (*P* < 0.0001). The body weights of mice in the two undernutrition groups showed no significant difference, and they were significantly lower than those of obesity and normal mice (*P* < 0.0001). At the 5th week after establishing undernutrition and obesity mouse models, the spleen weight decreased significantly in undernutrition 75% mice (*P* = 0.0145) and undernutrition 65% mice (*P* = 0.0148). The livers of obesity mice were obviously enlarged, and the weights were significantly heavier than those of the other groups. These results suggested that undernutrition and obesity mouse models were successfully established.

The amastigotes of *Leishmania* were found in spleen imprints of mice at the 8th week post-infection (Additional file 1: Fig. S2), and granulomatous lesions were observed on liver pathological sections (Additional file 1: Fig. S3). The number of granulomas was the highest in the infection control group and the least in the undernutrition 65% + infection group at the 5th week post-infection (Fig. 1B). Conversely, the number of granulomas in undernutrition 65% + infection group was the highest and in normal + infection and obesity + infection groups was the least at the 8th week post-infection. Furthermore, obvious splenomegaly and increased spleen weight were observed in undernutrition 65% + infection group at the 8th week post-infection (Fig. 1C). In undernutrition 75% + infection group, the number of granulomas was less, and splenomegaly was not significant. Therefore, among the infected groups, undernutrition 75% + infection group rather than undernutrition 65% + infection group showed the highest spleen and liver parasite load at the 8th week after infection (Fig. 1D). The spleen parasite load was the lowest in obesity + infection mice, and the liver parasite load was the lowest in normal + infection mice. These results showed that two undernutrition infected groups developed liver granulomas later, more serious splenomegaly and higher parasite load in the spleen, which caused more severe leishmaniasis.

### Expression of T cell exhaustion, T cell activation, apoptosis and Toll-like receptor genes

**Fig. 2:**
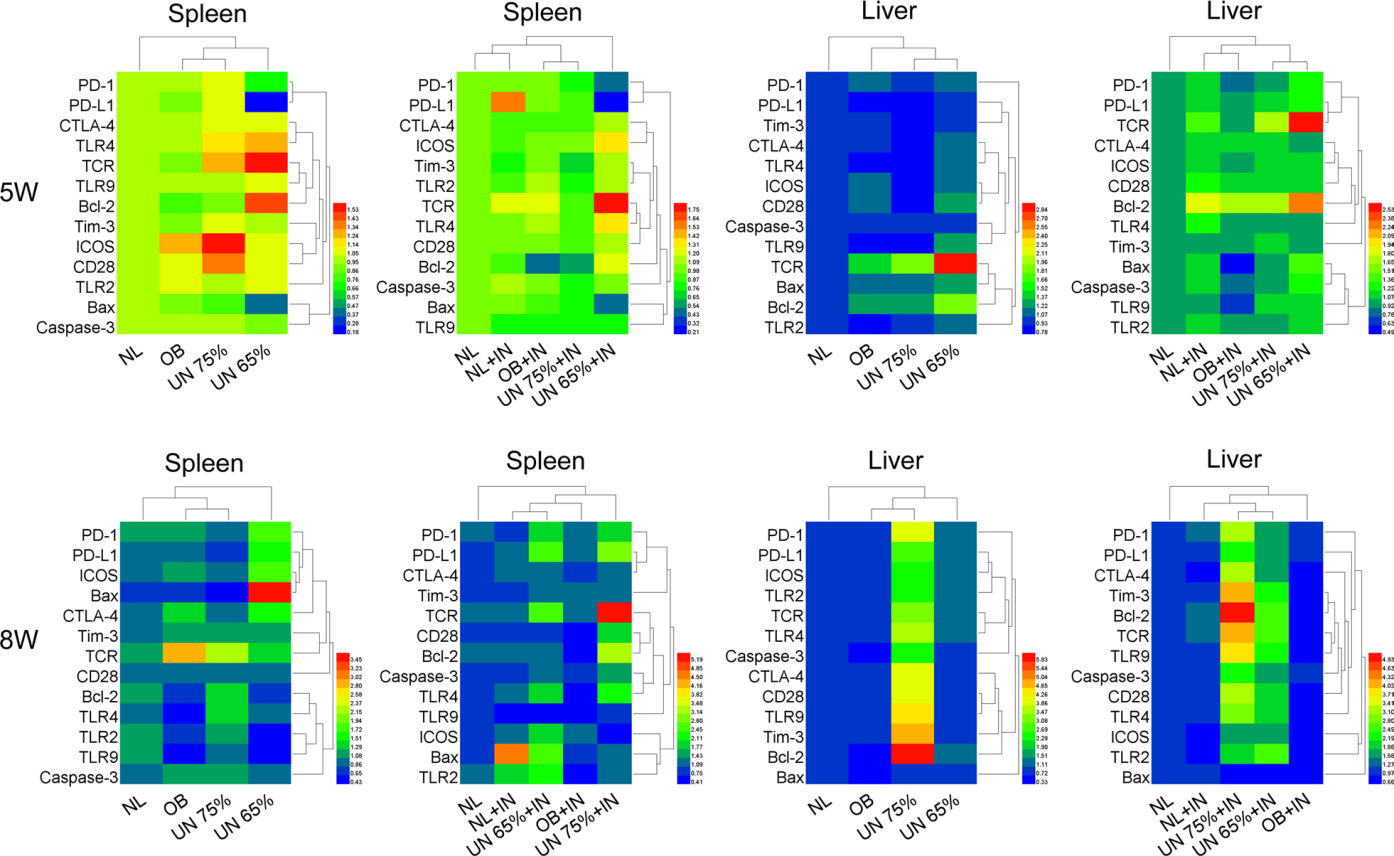
Expression heat map of T cell exhaustion, T cell activation, apoptosis and Toll-like receptor genes. IN: infection; NL: normal mice; OB: obesity mice; UN: undernutrition mice. The relative expression of genes in spleen and liver of different model mice was detected by qPCR at the 5th and 8th weeks after *Leishmania* infection. In the heat map, red represents high expression, and blue represents low expression

As shown in Fig. 2, the expression results of T cell exhaustion, T cell activation, apoptosis and Toll-like receptor genes in the spleen and liver were evaluated at the 5th and 8th weeks after infection. In the spleen, there were no obvious changes in the expression of each gene in undernutrition 75% + infection group, and only the apoptosis inhibiting gene *Bcl-2* (*P* = 0.0344) and T cell exhaustion gene *Tim-3* (*P* = 0.0494) were downregulated at the 5th week post-infection. In the undernutrition 65% + infection group, the T cell exhaustion gene *PD-1* (*P* = 0.0312) and *PD-L1* (*P* = 0.0016) as well as the apoptosis promoting gene *Bax* (*P* = 0.0152) were obviously downregulated. Meanwhile, the T cell activation gene *TCR* (*P* = 0.0415), apoptosis inhibiting gene *Bcl-2* (*P* = 0.0229) and *TLR4* (*P* = 0.0302) were upregulated; especially *TCR* was upregulated the most. However, at the 8th week post-infection, *PD-1* (*P* = 0.0420), *PD-L1* (*P* = 0.0406), *TCR* (*P* = 0.0423), *Bcl-2* (*P* = 0.0178), *Caspase-3* (*P* = 0.0349) and *TLR4* (*P* = 0.0449) were upregulated in undernutrition 75% + infection group. In undernutrition 65% + infection group, *PD-1* (*P* = 0.0364), *PD-L1* (*P* = 0.0481) and *Bax* (*P* = 0.0394) were conversely upregulated, and *ICOS* (*P* = 0.0274), *TLR2* (*P* = 0.0097) and *TLR4* (*P* = 0.0361) were also upregulated. These results indicated that the spleen of mice in undernutrition 75% + infection group did not show any obvious immune activation at the 5th week post-infection, but both immune activation and inhibitory genes were upregulated at the 8th week post-infection. Moreover, the spleen of mice in undernutrition 65% + infection group showed immune activation and decreased apoptosis at the 5th week post-infection, while the spleen was in a state of immune exhaustion and increased apoptosis at the 8th week post-infection.

In the liver, *CTLA-4* (*P* = 0.0473), *TCR* (*P* = 0.0470), *ICOS* (*P* = 0.0382), *CD28* (*P* = 0.0072) and *Bcl-2* (*P* = 0.0462) were upregulated in undernutrition 75% + infection group at the 5th week post-infection. In undernutrition 65% + infection group, *PD-1* (*P* = 0.0231), *TCR* (*P* = 0.0075), *Bcl-2* (*P* = 0.0110) and *Caspase-3* (*P* = 0.0475) were all upregulated, and *TCR* was also upregulated the most. Similarly, gene expression changes were seen at the 8th week post-infection. In undernutrition 75% + infection group, except for *Bax*, the rest of the genes were upregulated; especially *Bcl-2*, *Tim-3* and *TCR* were upregulated the most. The expression results of each gene in undernutrition 65% + infection group were similar to those in undernutrition 75% + infection group, but the upregulation was not as much as that in undernutrition 75% + infection group. These results suggested that the livers of mice in two undernutrition infected groups showed simultaneous upregulation of immune activation signals and inhibition signals at the 5th week after infection which continued to the 8th week after infection. Furthermore, *PD-1*, *PD-L1* and *TCR* were more upregulated in undernutrition 65% + infection group at the 5th week post-infection, while *PD-1*, *PD-L1* and *TCR* were more upregulated in undernutrition 75% + infection group at the 8th week post-infection.

### The proportions of CD3^+^, CD3^+^CD4^+^, CD3^+^CD8^+^ T cells and their PD-1 expression

**Fig. 3:**
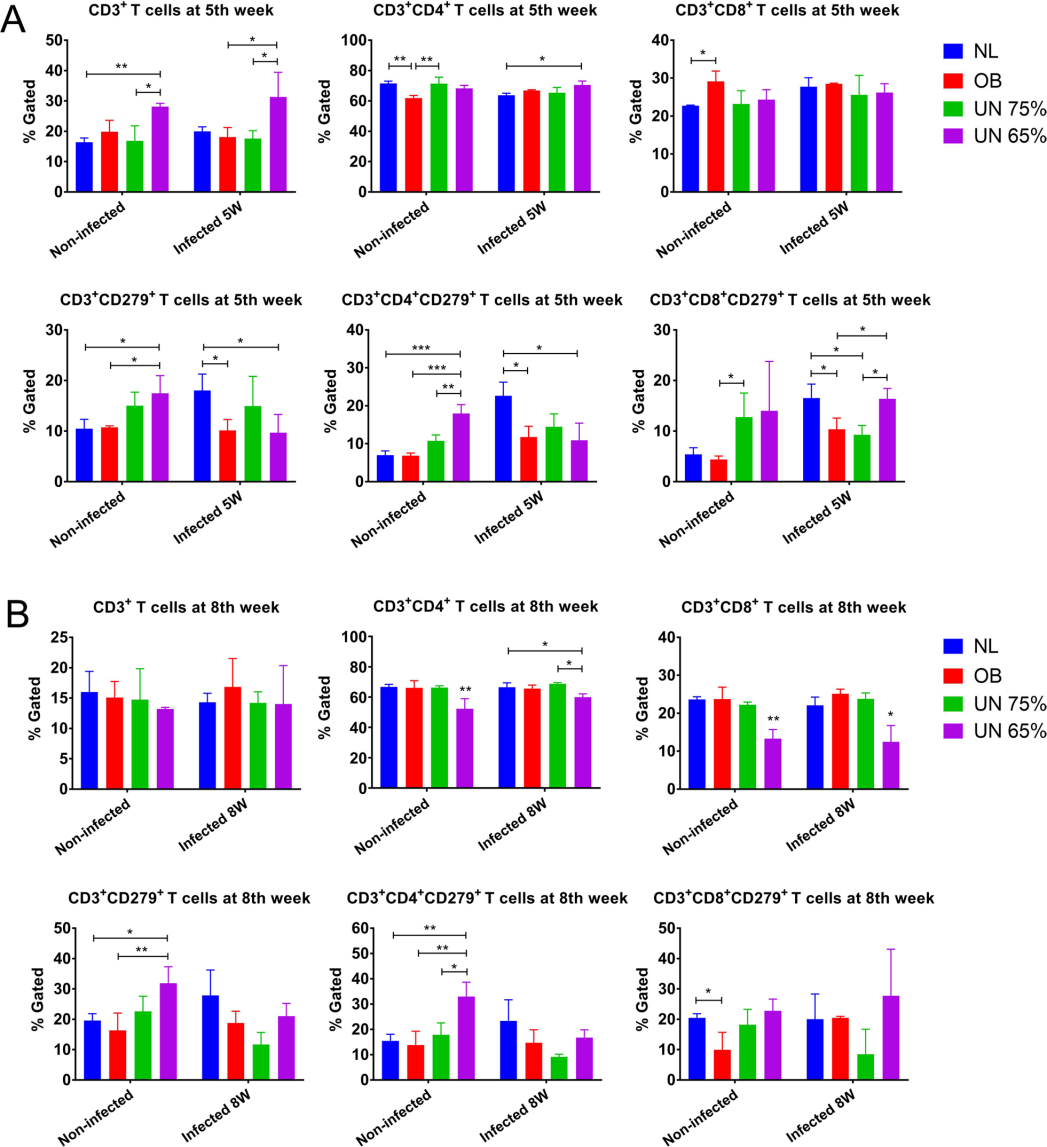
Flow cytometry detection of spleen lymphocyte subset and PD-1 (CD279) expression in mice. NL: normal mice; OB: obesity mice; UN: undernutrition mice. **A** Proportions of CD3^+^, CD3^+^CD4^+^, CD3^+^CD8^+^, CD3^+^CD279^+^, CD3^+^CD4^+^CD279^+^ and CD3^+^CD8^+^CD279^+^ T cells in spleen at the 5th week post-infection. **B** Proportions of CD3^+^, CD3^+^CD4^+^, CD3^+^CD8^+^, CD3^+^CD279^+^, CD3^+^CD4^+^CD279^+^ and CD3^+^CD8^+^CD279^+^ T cells in spleen at the 8th week post-infection

The analysis and statistical results for T lymphocytes detected by flow cytometry are shown in Fig. 3, and flow gating of CD3^+^CD279^+^, CD3^+^CD4^+^CD279^+^ and CD3^+^CD8^+^CD279^+^ T cells is shown in Additional file 1: Fig. S4. The proportions of CD3^+^ (*P* = 0.0324) and CD3^+^CD4^+^ (*P* = 0.0305) T cells in the spleens of mice from undernutrition 65% + infection group were significantly higher than those in normal + infection group or obesity + infection group at the 5th week after infection, but the proportions of CD3^+^CD4^+^ (*P* = 0.0483) and CD3^+^CD8^+^ (*P* = 0.0307) T cells were significantly lower at the 8th week after infection (Fig. 3A, B). The expression of CD279 (PD-1) on CD3^+^, CD3^+^CD4^+^ and CD3^+^CD8^+^ T cells was upregulated in normal + infection mice, consistent with reports from da Fonseca-Martins et al. [11] and Habib et al. [12]. In uninfected groups, compared with the normal group, the proportions of CD3^+^CD279^+^, CD3^+^CD4^+^CD279^+^ and CD3^+^CD8^+^CD279^+^ T cells were increased in two undernutrition groups. However, in infected groups, compared with the normal + infection group, the proportions of CD3^+^CD279^+^ and CD3^+^CD4^+^CD279^+^ T cells were decreased in two undernutrition infected groups. These results indicated that undernutrition could affect the proportions of T lymphocytes and upregulate PD-1 expression in CD3^+^, CD3^+^CD4^+^ and CD3^+^CD8^+^ T cells. However, PD-1 expression in CD3^+^ and CD3^+^CD4^+^ T cells was downregulated under the combined influence of undernutrition and *Leishmania* infection.

### Serum anti-*Leishmania* total IgG, IgG1 and IgG2a antibodies

**Fig. 4:**
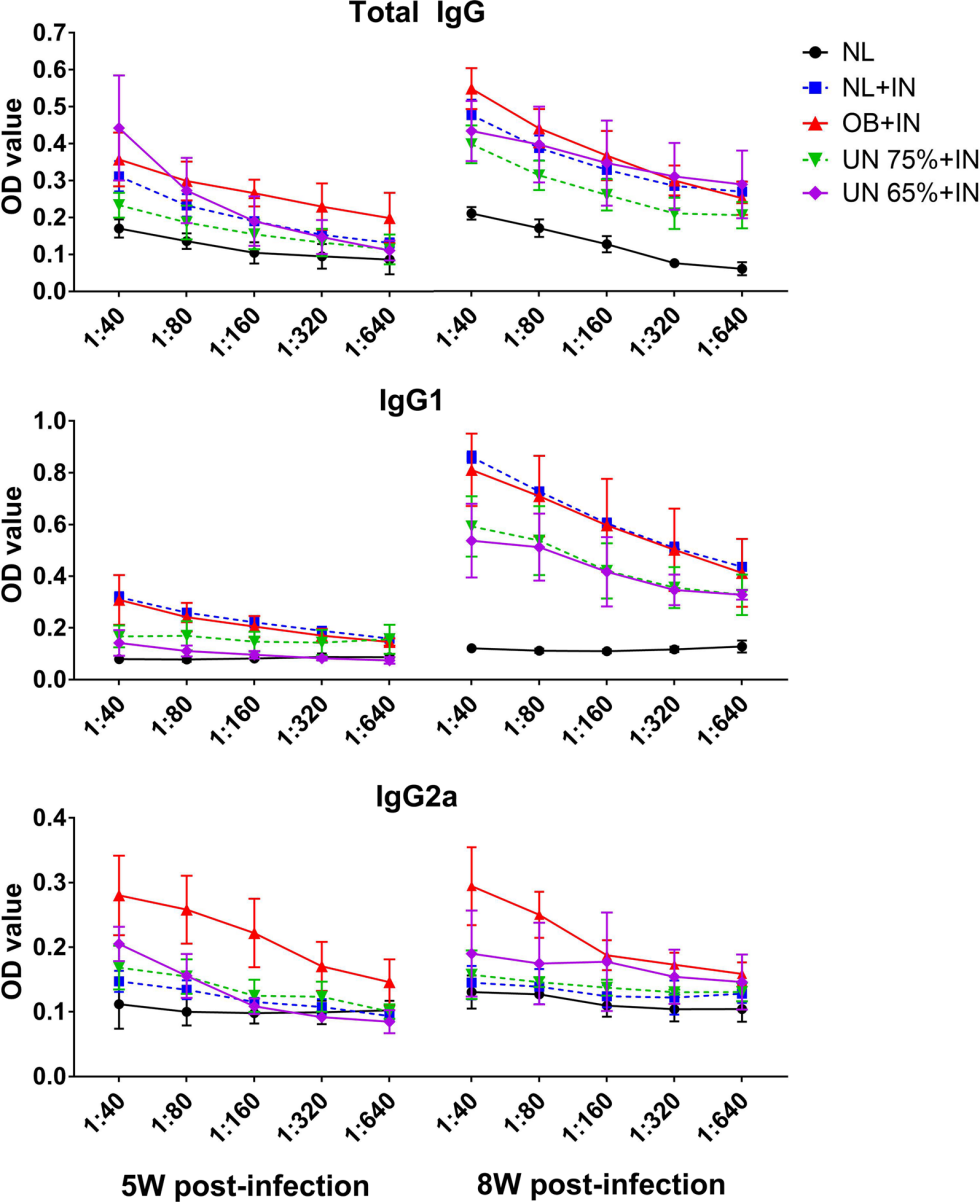
Anti-*Leishmania* IgG, IgG1 and IgG2a antibody titers in the serum of mice after infection. IN: infection; NL: normal mice; OB: obesity mice; UN: undernutrition mice. The sera of uninfected normal mice were used as negative controls

The OD values of anti-*Leishmania* total IgG, IgG1 and IgG2a antibodies in the serum of infected mice at different dilutions are shown in Fig. 4. The OD values of total IgG and IgG1 in each group at the 8th week after infection were significantly higher than those at the 5th week after infection, indicating the antibody titers increase with the infection time in the early stage of leishmaniasis. At the 5th and 8th weeks after infection, the OD values of total IgG in undernutrition 75% + infection group were the lowest under different dilutions. The OD values of IgG1 were the lowest in undernutrition 65% + infection group at the 5th week post-infection and the lowest in two undernutrition infected groups at the 8th week post-infection. The OD values of IgG2a were the lowest in undernutrition 75% + infection and normal + infection groups. These results were basically consistent with the results of parasite load in the spleen and liver. The OD values of IgG, IgG1 and IgG2a antibodies were the lowest in undernutrition 75% + infection group, and the parasite load of this group was the highest at the 8th week post-infection.

### Serum lipids, transaminases and cytokines

**Fig. 5:**
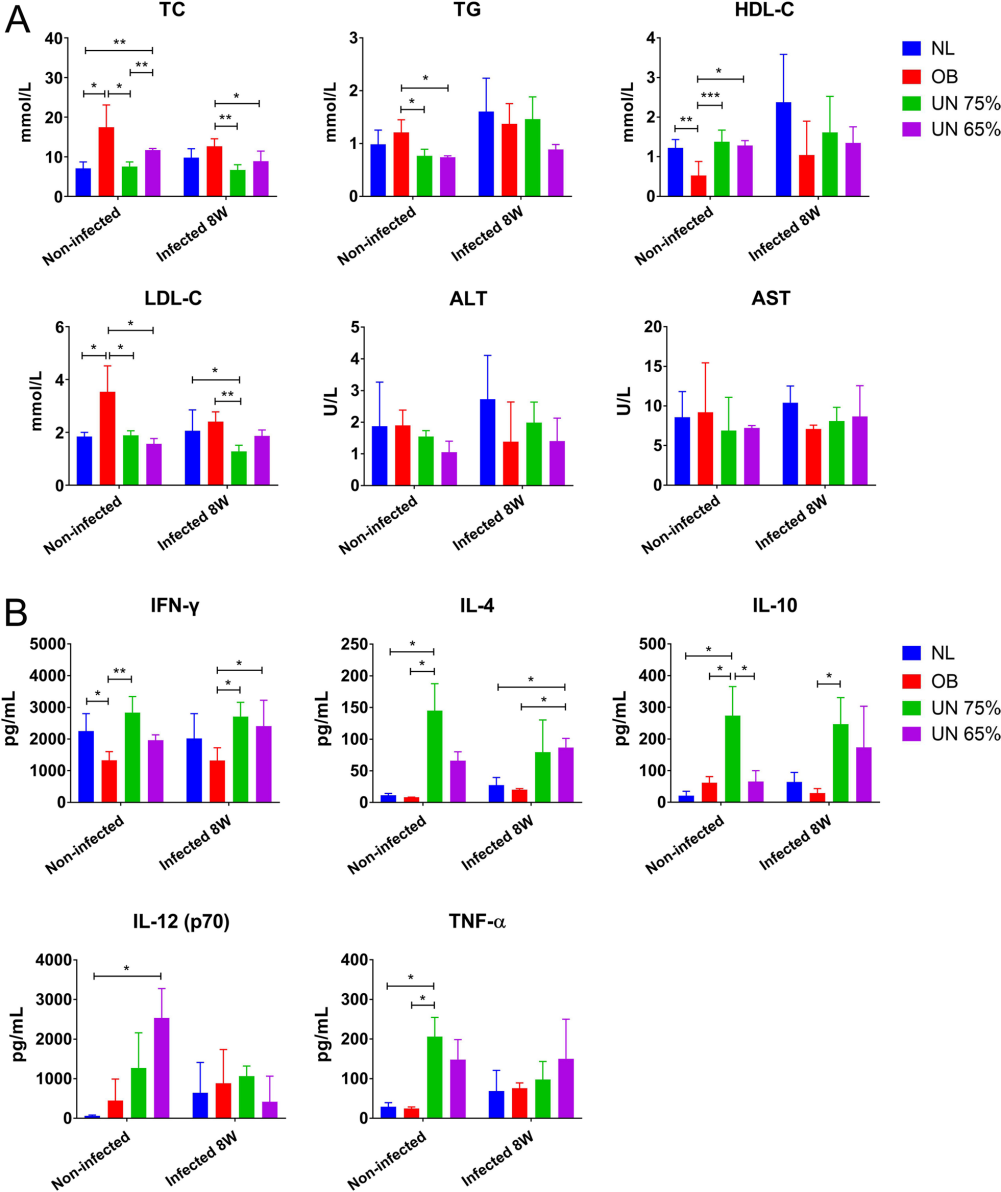
Serum lipid, transaminase and cytokine levels of mice at the 8th week after *Leishmania* infection. NL: normal mice; OB: obesity mice; UN: undernutrition mice. **A** Serum lipid and transaminase levels of mice in each group at the 8th week post-infection. **B** The levels of serum cytokines IFN-γ, TNF-α, IL-4, IL-10 and IL-12 p70 in mice at the 8th week post-infection

Studies have found that *Leishmania* infection affects the lipid metabolism of the host [28, 29]. Therefore, we detected the serum lipid levels of mice in each group, and the results are shown in Fig. 5A. In uninfected groups, the levels of TC, TG and LDL-C in obesity group were significantly higher than those in other groups, and the level of HDL-C was significantly lower than that in other groups. The results of serum TG, LDL-C and HDL-C in two undernutrition groups were not statistically different from those in normal group. After infection, serum TC (*P* = 0.0353), TG (*P* = 0.0370) and HDL-C (*P* = 0.0433) increased in normal group, providing evidence of lipid metabolism changes caused by *Leishmania* infection. However, TC (*P* = 0.0296) and LDL-C (*P* = 0.0454) decreased in obesity group, while LDL-C (*P* = 0.0004) decreased in undernutrition 75% group after infection. The results of serum ALT and AST in each group showed no statistical difference (Fig. 5A).

The changes of serum cytokines in each group after infection were shown in Fig. 5B. In uninfected groups, two undernutrition groups showed different serum cytokine levels from those of normal group. The levels of TNF-α (*P* = 0.0145), IL-4 (*P* = 0.0118) and IL-10 (*P* = 0.0224) in undernutrition 75% group were significantly higher than those in normal group, and the level of IL-12 p70 (*P* = 0.0206) in undernutrition 65% group was significantly higher than that in normal group. These results demonstrated that some inflammatory factors had been activated in the bodies of undernutrition mice. After infection, the results of IFN-γ, TNF-α, IL-4, IL-10 and IL-12 p70 in obesity + infection group were not obviously different from those in normal + infection group. The levels of IFN-γ, IL-4 and IL-10 in two undernutrition infected groups were higher than those in normal + infection and obesity + infection groups, while the results of TNF-α and IL-12 p70 were not different among the groups.

### Analysis and clustering of differentially expressed genes in spleen transcriptome

**Fig. 6:**
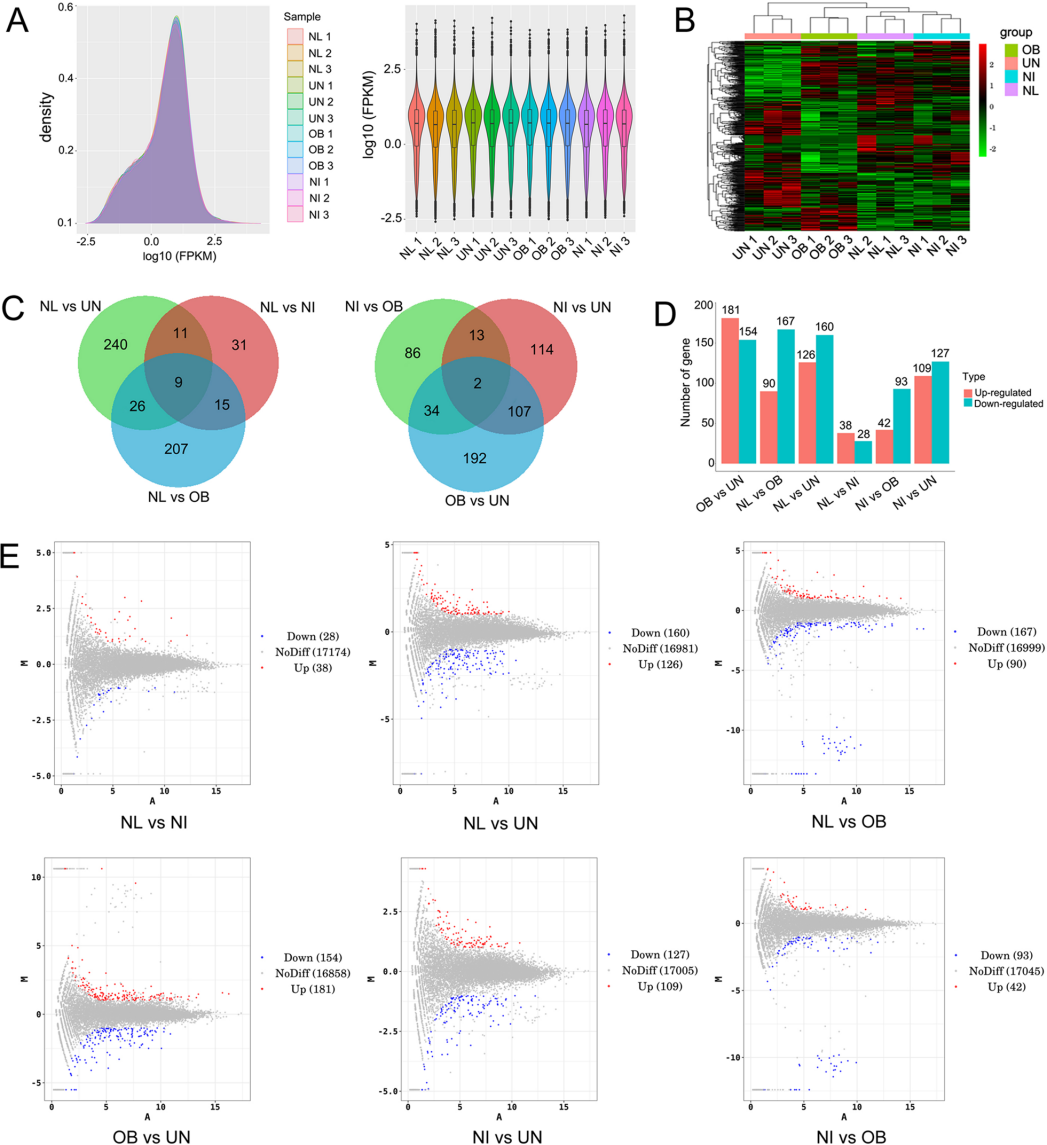
Transcriptome sequencing of spleen tissues to find differentially expressed genes at the 8th week post-infection. NL: normal group; NI: normal + infection group; OB: obesity + infection group; UN: undernutrition 75% + infection group. **A** FPKM density distribution and violin plots for all samples. In violin plots, the horizontal line in the middle of the box is the median, the upper and lower edges of the box are at 75%; the upper and lower limits are at 90%. **B** Clustering of DEGs in all samples. Each column is a sample, and the horizontal axis represents different genes, with red representing high expression genes and green representing low expression genes. **C** Venn diagram of DEGs in each group. **D** The number of DEGs in the pairwise comparison of each group. Red represents upregulated genes, and green represents downregulated genes. **E** MA plots of DEGs between groups. Red dots represent upregulated genes, blue dots represent downregulated genes, and gray dots represent non-differentially expressed genes compared with the control group

FPKM density distribution and violin plots for all samples are shown in Fig. 6A, and the expression pattern of each sample was basically the same. As shown in Fig. 6B, all samples were clustered based on DEGs, and the results demonstrated that the biological replicates of each group clustered well together and the gene expression differed significantly among groups. The number of DEGs between groups is shown in Fig. 6C, D. Only nine genes were differentially expressed between the normal group and the infection groups, and two genes were differentially expressed among the three infection groups. The largest number of DEGs was found between obesity + infection group and undernutrition 75% + infection group, with 181 genes upregulated and 154 genes downregulated. MA graphs showed the distribution of DEGs among different groups (Fig. 6E). The number of upregulated DEGs was the largest between obesity + infection group and undernutrition 75% + infection group. The number of downregulated DEGs was the largest between normal group and obesity + infection group.

### KEGG signaling pathway and GSEA analysis

**Fig. 7:**
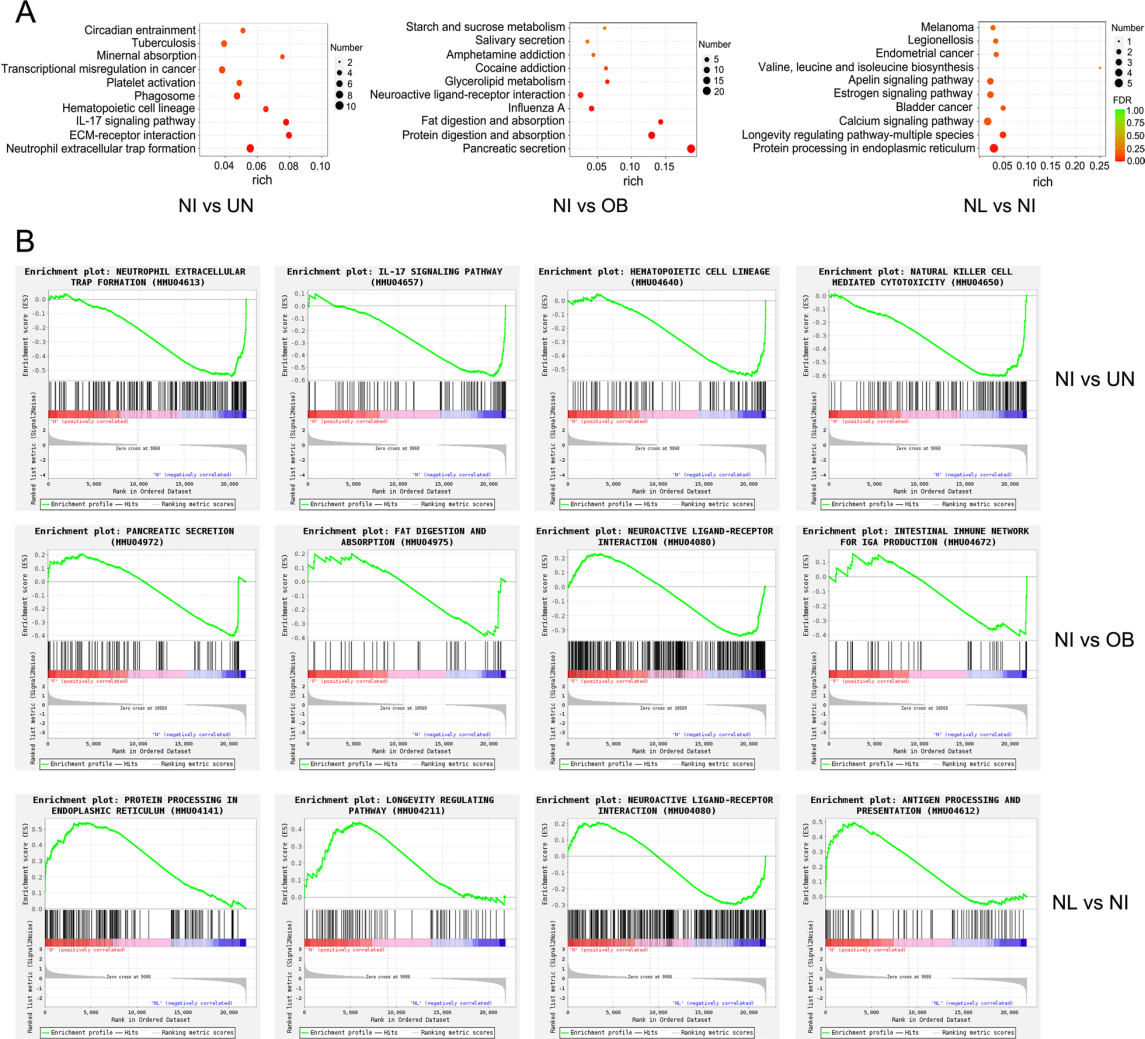
KEGG and GSEA enrichment analysis of differentially expressed genes. NL: normal group; NI: normal + infection group; OB: obesity + infection group; UN: undernutrition 75% + infection group. **A** The top 10 KEGG pathways with the most significant enrichment. The degree of enrichment was measured by the rich factor, FDR (false discovery rate) value and the number of genes enriched in the pathway. The greater the rich factor was, the greater the degree of enrichment. FDR generally ranges from 0 to 1, and the closer it is to zero, the more significant the enrichment is. The larger the bubble diameter in the figure, the more differentially expressed genes enriched in the pathway. **B** GSEA analysis results of important signaling pathways

The enriched top three KEGG signaling pathways of DEGs in normal + infection and undernutrition 75% + infection groups were neutrophil extracellular trap formation (*P* < 0.0001), ECM-receptor interaction (*P* < 0.0001) and IL-17 signaling pathway (*P* < 0.0001) (Fig. 7A). The results of GSEA analysis showed that the expression of gene sets in neutrophil extracellular trap formation (*P* < 0.0001), IL-17 signaling pathway (*P* < 0.0001), hematopoietic cell lineage (*P* < 0.0001) and natural killer cell-mediated cytotoxicity (*P* < 0.0001) was higher in normal + infection group and lower in undernutrition 75% + infection group (Fig. 7B). Eleven genes in neutrophil extracellular trap formation were downregulated in undernutrition 75% + infection group, which was the signaling pathway with the largest number of downregulated genes. In normal + infection and obesity + infection groups, the enriched top three KEGG pathways were pancreatic secretion (*P* < 0.0001), protein digestion and absorption (*P* < 0.0001), and fat digestion and absorption (*P* < 0.0001). GSEA results showed that the expression of gene sets in pancreatic secretion (*P* = 0.0040), fat digestion and absorption (*P* = 0.0152), neuroactive ligand-receptor interaction (*P* < 0.0001) and intestinal immune network for IgA production (*P* = 0.0159) was slightly higher in normal + infection group and lower in obesity + infection group. In normal and normal + infection groups, the enriched top three KEGG pathways were protein processing in endoplasmic reticulum (*P* = 0.0001), longevity regulating pathway (*P* = 0.0007) and calcium signaling pathway (*P* = 0.0049). GSEA results showed that the expression of gene sets in protein processing in endoplasmic reticulum (*P* < 0.0001), longevity regulating pathway (*P* = 0.0090) and antigen processing and presentation (*P* = 0.0020) was higher in normal + infection group and lower in normal group.

### PPI analysis and screening of hub genes

**Fig. 8:**
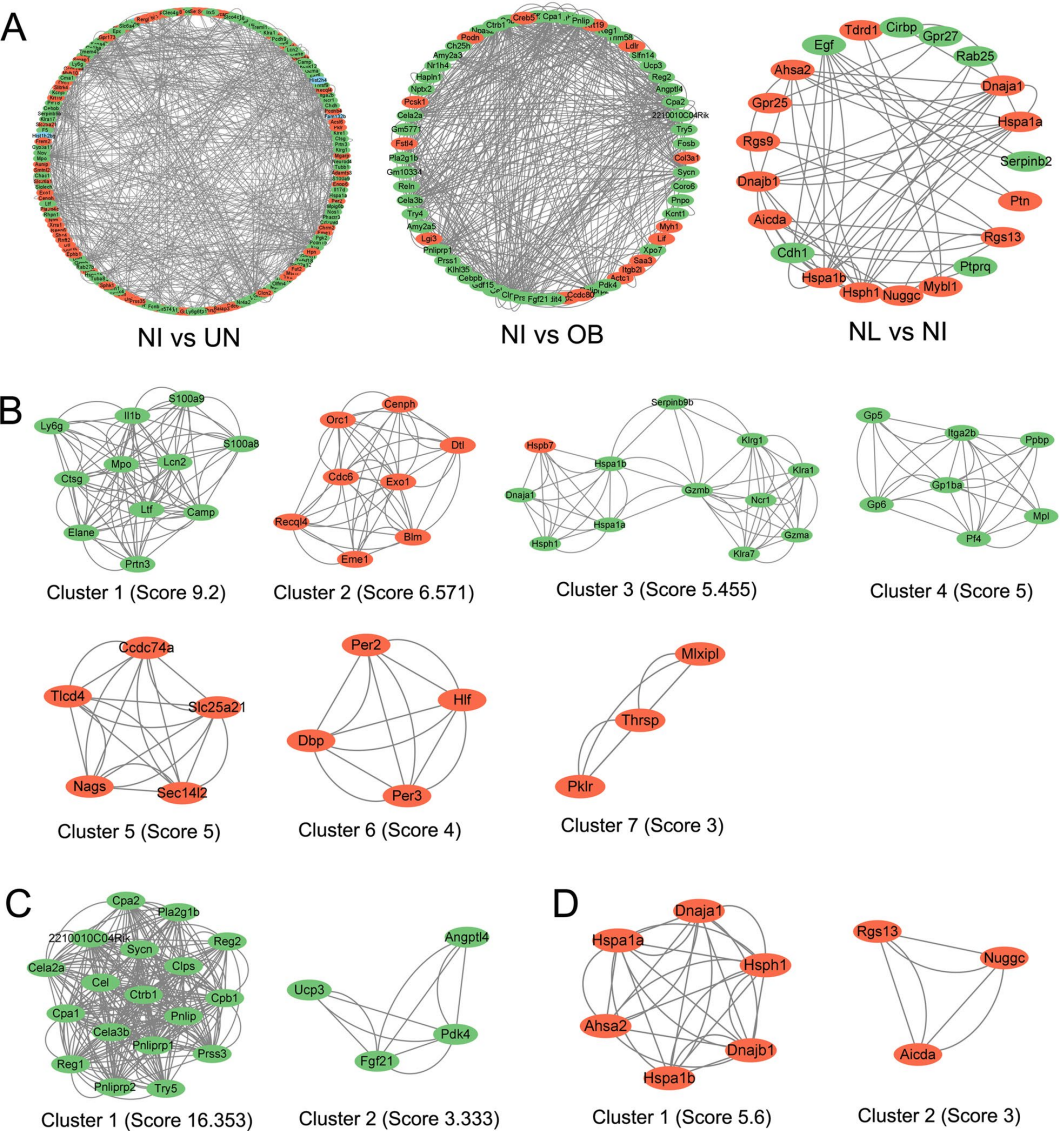
PPI analysis of differentially expressed genes and screening of hub genes. NL: normal group; NI: normal + infection group; OB: obesity + infection group; UN: undernutrition 75% + infection group. Each ellipse in the figure represents a protein. Red represents upregulated expression, and green represents downregulated expression compared with the control group. **A** PPI interaction circular map of DEGs. **B** Hub gene clusters of DEGs between normal + infection and undernutrition 75% + infection groups. **C** Hub gene clusters of DEGs between normal + infection and obesity + infection groups. **D** Hub gene clusters of DEGs between normal and normal + infection group

The PPI networks of DEGs are shown in Fig. 8A. Hub gene clusters and scores of DEGs between normal + infection and undernutrition 75% + infection groups were shown in Fig. 8B, with a total of seven clusters. Clusters 1, 3 and 4 were downregulated, and other clusters were upregulated. The KEGG pathways enriched by gene clusters are shown in Additional file 2: Table S1. The signaling pathways enriched by the three downregulated clusters were basically consistent with the results of KEGG and GSEA analysis. Therefore, we thought that a total of 30 hub genes in clusters 1, 3 and 4 in undernutrition 75% + infection group might play important roles in the interaction between undernutrition and *Leishmania* infection. Hub gene clusters of DEGs between normal + infection and obesity + infection groups are shown in Fig. 8C. There were only two clusters, and both were downregulated. According to the results of KEGG pathway enriched by these two clusters, 18 hub genes in cluster 1 were considered crucial in the interaction between obesity and *Leishmania* infection. As shown in Fig. 8D, two hub gene clusters of DEGs were found in normal and normal + infection groups, and both were upregulated. Six hub genes on cluster 1 were thought to be important in *Leishmania* infection. The functions of all the important hub genes in the UniProt database are shown in Additional file 2: Table S2.

## Discussion

Undernutrition is associated with many problems including fatigue, anemia, immune dysfunctions and increased susceptibility to infections [30]. In undernutrition patients, both innate and acquired immunity are affected, and common immune defects include CD4/CD8 T cells ratio imbalance, reduced antibody responses, impaired phagocytosis of macrophages and decreased NF-kappaB activation by macrophages [31, 32]. In this study, we focused on evaluating the progress of leishmaniasis in the spleen and liver, the expression of immunosuppressive molecules PD-1 and PD-L1, and their relationships with T cell activation and apoptosis in *Leishmania*-infected mice with different nutritional imbalances. The possible molecular mechanisms of undernutrition promoting leishmaniasis were revealed using RNA-seq of spleen tissues, and the signaling pathways enriched by DEGs and hub genes were screened.

T cell exhaustion is known to be driven by a high level of sustained TCR signaling [33, 34], and exhaustion is considered a means of carefully balancing control of chronic infection with the risk of damaging immunopathology [35]. During acute T cell activation, strong TCR signals drive Akt-mediated cytotoxicity and PD-1 expression to establish a feedback loop, which limits TCR signaling [36, 37]. However, if TCR signals persist, they will be desensitized by PD-1, which will allow maintaining a longer term but more restrained T cell response [36, 37]. In this study, at the 5th week after infection, the parasite load of undernutrition 65% + infection group in the spleen was the lowest, the expression of *PD-1*, *PD-L1* and *Bax* in the spleen of this group was downregulated, and *TCR*, *Bcl-2* and *TLR4* were upregulated. The proportions of CD3^+^ and CD3^+^CD4^+^ T cells were increased, and CD3^+^CD279^+^ and CD3^+^CD4^+^CD279^+^ T cells were decreased in this group. These results suggested that the immune system was activated in undernutrition 65% + infection group at the 5th week post-infection, with reduced T cell exhaustion and cell apoptosis. At the 8th week post-infection, in undernutrition 65% + infection group, *PD-1*, *PD-L1*, *Bax* and *ICOS* were upregulated and CD3^+^CD4^+^ and CD3^+^CD8^+^ T cell proportions were decreased, while the level of serum IL-10 was increased and IL-12 p70 was decreased. These results suggested that the immune system was suppressed in this group at the 8th week after infection, T cell exhaustion and cell apoptosis were increased. There was no obvious immune activation in the spleen of undernutrition 75% + infection group at the 5th week post-infection. However, at the 8th week post-infection, *PD-1*, *PD-L1*, *Bcl-2*, *Caspase-3* and *TCR* in the spleen of this group were upregulated, and the values of total IgG, IgG1 and IgG2a were all low, indicating that this might be due to immunosuppression and increased T cell exhaustion resulting in the highest parasite load. At the 8th week post-infection in the liver, *PD-1*, *PD-L1*, *CTLA-4*, *Tim-3*, *Caspase-3* and *TCR* were all upregulated in two undernutrition infected groups, but more upregulated in undernutrition 75% + infection group, which might explain why the parasite load of undernutrition 75% + infection group was also the highest in the liver.

The results of KEGG and GSEA analysis showed that some signaling pathways were downregulated in undernutrition 75% + infection group, including neutrophil extracellular trap formation, IL-17 signaling pathway, hematopoietic cell lineage, natural killer cell mediated cytotoxicity, etc. In particular, 11 genes were downregulated in neutrophil extracellular trap formation, which was the signaling pathway with the largest number of downregulated genes. Neutrophil extracellular traps (NETs) consist of a DNA backbone along with microbicidal proteins and can be released to kill pathogens after encountering bacterial or fungal infection [38]. The interaction of *Leishmania* with human neutrophils leads to the release of NETs, which traps and kills the parasite [39]. Therefore, the downregulation of these immune signaling pathways might be one of the important reasons why undernutrition mice developed more severe VL.

In the PPI network, the central genes of cluster 1 between normal + infection group and undernutrition 75% + infection group were *Mpo* (0.31 fold change), *Lcn2* (0.38 fold change) and *Ltf* (0.28 fold change), which were downregulated in undernutrition 75% + infection group. Myeloperoxidase (Mpo) is a part of polymorphonuclear leukocytes and responsible for microbicidal activity against a wide range of organisms [40], which participate in neutrophil extracellular trap formation [41] and phagosome [42]. Neutrophil gelatinase-associated lipocalin (Lcn2) is an iron-trafficking protein involved in multiple processes such as apoptosis, innate immunity and renal development [43]. Moreover, Lcn2 and S100A family members such as S100a8 and S100a9 all participate in IL-17 signaling pathway [44, 45]. Lactotransferrin (Ltf) is a major iron-binding and multifunctional protein found in exocrine fluids, which has antimicrobial activity and may interfere with lipopolysaccharide (LPS)-stimulated TLR4 signaling [46]. Consequently, the decreased expression of these genes may inhibit immune functions in the body. Interestingly, in cluster 3 of DEGs between normal + infection group and undernutrition 75% + infection group, hub genes *Dnaja1* (0.49-fold change), *Hspa1a* (0.12-fold change), *Hspa1b* (0.20-fold change) and *Hsph1* (0.35-fold change) were also downregulated in undernutrition 75% + infection group. These hub genes belong to heat shock protein (HSP) genes and participate in a wide variety of cellular processes, including protecting the proteome from stress, ensuring the correct folding of proteins and controlling the targeting of proteins for subsequent degradation [47]. Recent studies have found that HSPs are involved in immunomodulation, and some Hsps are implicated in antigen processing and presentation [48, 49]. Therefore, the downregulation of these HSP genes may also be one of the reasons for the immunosuppression in undernutrition 75% + infection group.

## Conclusions

In this study, two undernutrition infected groups showed more severe leishmaniasis, with increased parasite load in the spleen, upregulated expression of *PD-1* and *PD-L1*, elevated serum IL-4 and IL-10, and decreased serum antibodies, TC and LDL-C. Transcriptome sequencing analysis revealed that the immunosuppression in undernutrition 75% + infection group might be caused by the downregulation of immune signaling pathways such as neutrophil extracellular trap formation, IL-17 signaling pathway and natural killer cell-mediated cytotoxicity. Our results demonstrated that the mechanism of undernutrition promoting VL was related to the upregulation of PD-1/PD-L1 and the downregulation of immune signaling pathways, which provided a basis for studying the correlation between undernutrition and leishmaniasis. The immune signaling pathways and hub genes may serve as drug targets or intervention targets for the treatment of VL in undernutrition populations in the future.

### Supplementary Information

**Additional file 1. Fig. S1**: The amplification curve for the qPCR results. **Fig. S2**: Spleen tissue imprints of mice at the 8th week after *Leishmania* infection. Spleen tissue imprints were stained with Wright's stain and observed under a 100× objective lens (×1000). The red arrow points to *Leishmania* amastigotes. **A** Spleen tissue imprint of normal + infection mice. **B** Spleen tissue imprint of obesity + infection mice. **C** Spleen tissue imprint of undernutrition 75% + infection mice. **D** Spleen tissue imprint of undernutrition 65% + infection mice. **Fig. S3**: Pathological changes of the liver in model mice with different nutritional imbalance at the 5th and 8th weeks post-infection. Pathological sections were stained with hematoxylin-eosin and observed under a 40× objective lens (×400). Inflammatory lesions or granulomas in the liver were circled in white circles. The livers of obesity mice showed obvious fatty liver manifestations with fatty degeneration of hepatocytes, and the cells were swollen and filled with vacuoles of different sizes. **Fig. S4**: Flow cytometry gating of CD3^+^CD279^+^, CD3^+^CD4^+^CD279^+^ and CD3^+^CD8^+^CD279^+^ T cells. The histogram showed CD3^+^CD279^+^T cells in some uninfected samples at the 5th week post-infection.

**Additional file 2. Table S1**: KEGG signaling pathway enriched by hub gene clusters. **Table S2**: The function of important hub genes in UniProt database.

## Data Availability

The datasets supporting the conclusions of this article are included within the article and its additional files.

## Abbreviations

DEGs: Differentially expressed genes
GSEA: Gene set enrichment analysis
KEGG: Kyoto Encyclopedia of Genes and Genomes
PD-1: Programmed cell death protein 1
PD-L1: Programmed cell death 1 ligand 1
PPI: Protein–protein interaction
STRING: Search tool for the retrieval of interacting genes
VL: Visceral leishmaniasis
