# Supplementary material for: Upregulation of PD-1/PD-L1 and downregulation of immune signaling pathways lead to more severe visceral leishmaniasis in undernutrition mice
Source: Parasit Vectors. 2024 Jan 8;17:8. doi: 10.1186/s13071-023-06018-2 (PMC10773036; doi:10.1186/s13071-023-06018-2)
Supplement: Supplementary file 1 — Additional file 1. Fig. S1: The amplification curve for the qPCR results. Fig. S2: Spleen tissue imprints of mice at the 8th week after Leishmania infection. Spleen tissue imprints were stained with Wright's stain and observed under a 100× objective lens (×1000). The red arrow points to Leishmania amastigotes. A Spleen tissue imprint of normal + infection mice. B Spleen tissue imprint of obesity + infection mice. C Spleen tissue imprint of undernutrition 75% + infection mice. D Spleen tissue imprint of undernutrition 65% + infection mice. Fig. S3: Pathological changes of the liver in model mice with different nutritional imbalance at the 5th and 8th weeks post-infection. Pathological sections were stained with hematoxylin-eosin and observed under a 40× objective lens (×400). Inflammatory lesions or granulomas in the liver were circled in white circles. The livers of obesity mice showed obvious fatty liver manifestations with fatty degeneration of hepatocytes, and the cells were swollen and filled with vacuoles of different sizes. Fig. S4: Flow cytometry gating of CD3+CD279+, CD3+CD4+CD279+ and CD3+CD8+CD279+ T cells. The histogram showed CD3+CD279+T cells in some uninfected samples at the 5th week post-infection. [file 13071_2023_6018_MOESM1_ESM.doc]

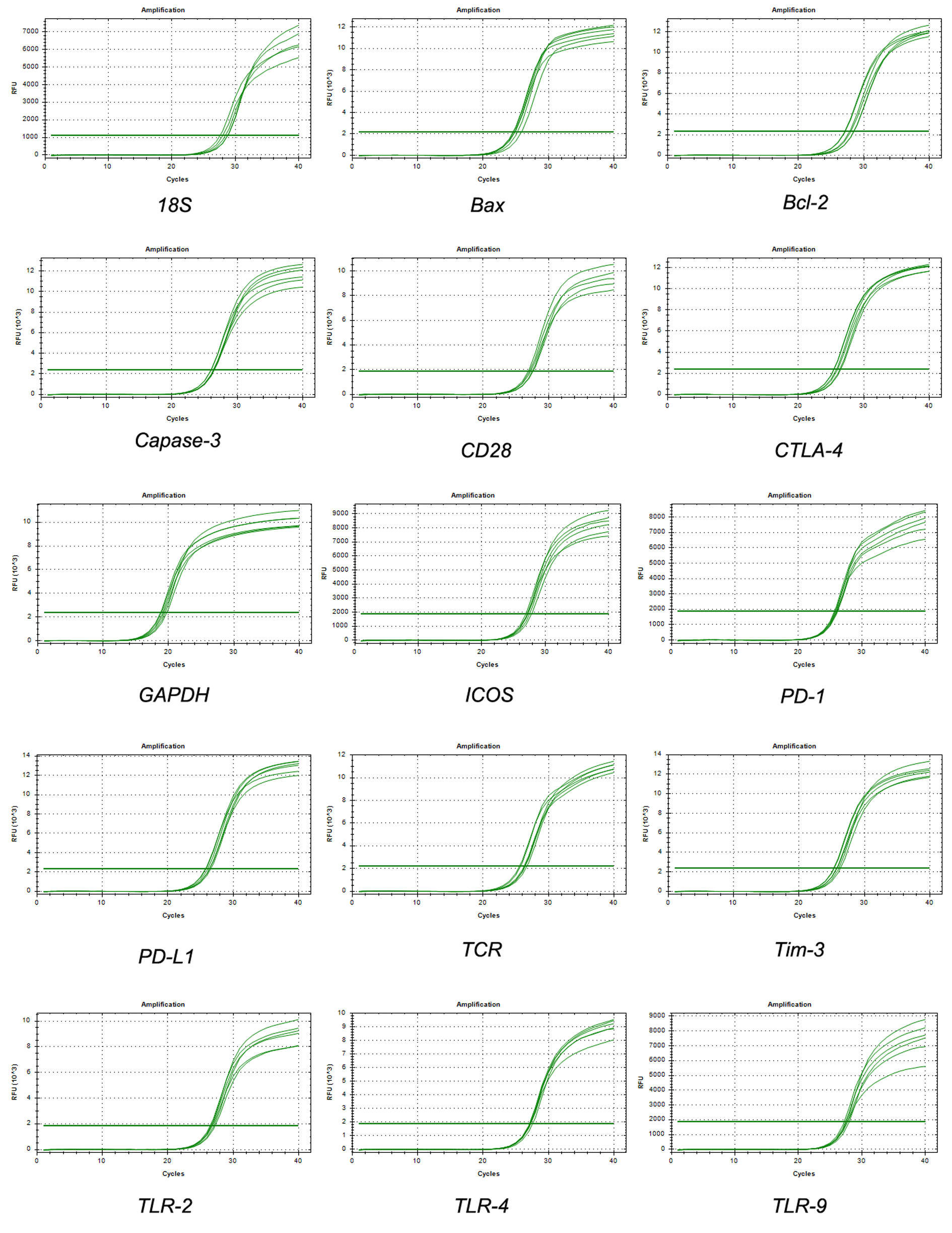


**Fig. S1** The amplification curve for the qPCR results.


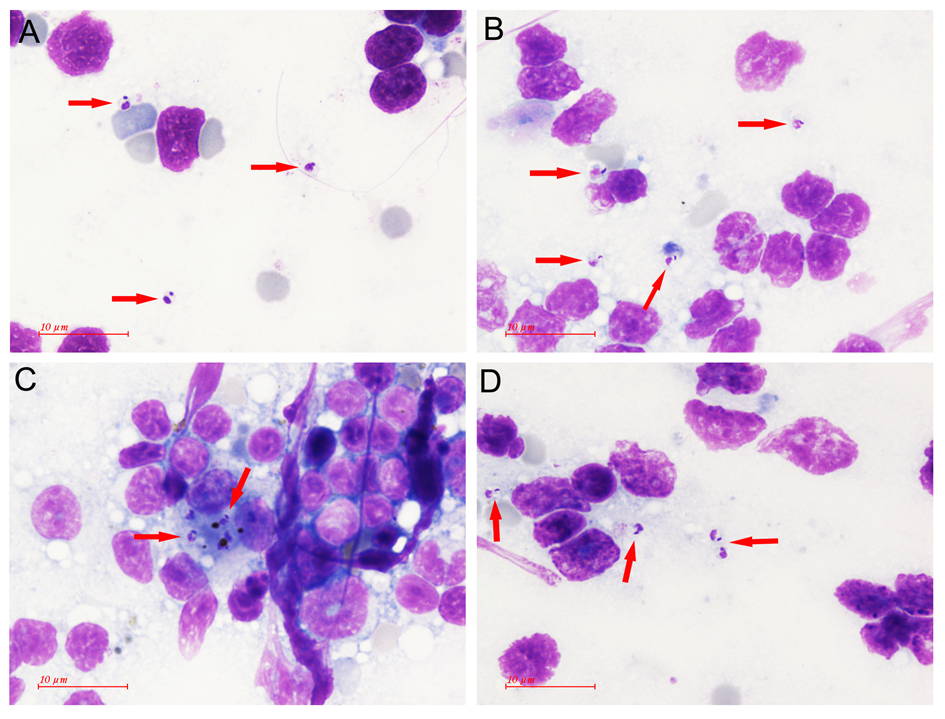


**Fig. S2** Spleen tissue imprints of mice at the 8th week after *Leishmania* infection. Spleen tissue imprints were stained with Wright's stain and observed under a 100× objective lens (×1000). The red arrow points to *Leishmania* amastigotes. **A** Spleen tissue imprint of normal+infection mice. **B** Spleen tissue imprint of fat+infection mice. **C** Spleen tissue imprint of undernutrition 75%+infection mice. **D** Spleen tissue imprint of undernutrition 65%+infection mice.


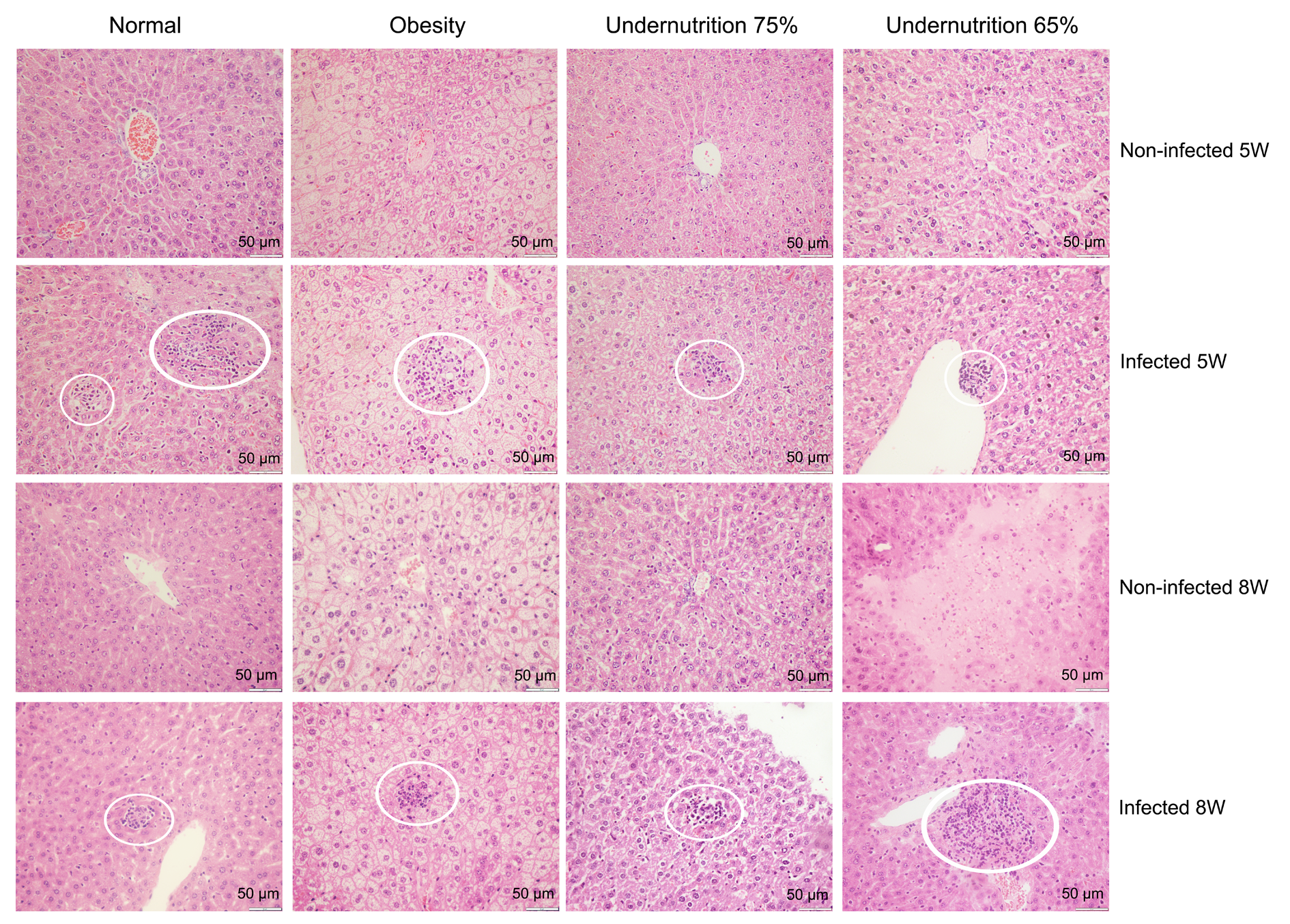


**Fig. S3** Pathological changes of the liver in model mice with different nutritional imbalance at the 5th and 8th weeks post-infection. Pathological sections were stained with hematoxylin-eosin and observed under a 40× objective lens (×400). Inflammatory lesions or granulomas in the liver were circled in white circles. The livers of obesity mice showed obvious fatty liver manifestations with fatty degeneration of hepatocytes, and the cells were swollen and filled with vacuoles of different sizes.


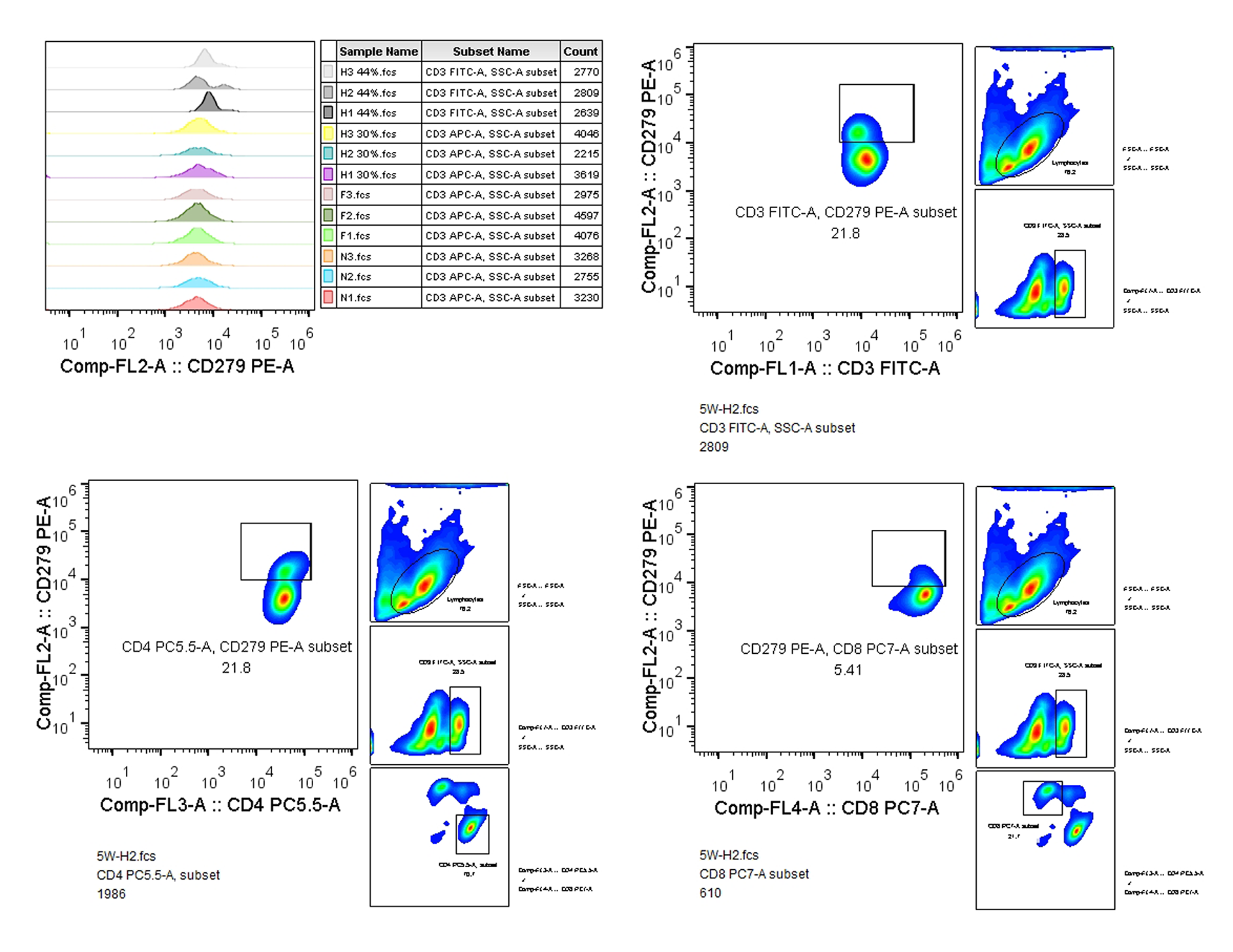


**Fig. S4** Flow cytometry gating of CD3^+^CD279^+^, CD3^+^CD4^+^CD279^+^ and CD3^+^CD8^+^CD279^+^ T cells. The histogram showed CD3^+^CD279^+^ T cells in some uninfected samples at the 5th week post-infection.
