# Supplementary material for: Upregulation of PD-1/PD-L1 and downregulation of immune signaling pathways lead to more severe visceral leishmaniasis in undernutrition mice
Source: Parasit Vectors. 2024 Jan 8;17:8. doi: 10.1186/s13071-023-06018-2 (PMC10773036; doi:10.1186/s13071-023-06018-2)
Supplement: Supplementary file 2 — Additional file 2. Table S1: KEGG signaling pathway enriched by hub gene clusters. Table S2: The function of important hub genes in UniProt database. [file 13071_2023_6018_MOESM2_ESM.doc]

**Table S1** KEGG signaling pathway enriched by hub gene clusters

| Groups | Cluster | Attribute | KEGG pathway | Strength |
| --- | --- | --- | --- | --- |
| NI vs UN | Cluster 1 | Down | IL-17 signaling pathway (mmu04657) | 1.95 |
|  | Cluster 2 | Up | Homologous recombination (mmu03440)  Fanconi anemia pathway (mmu03460) | 2.15  2.05 |
|  | Cluster 3 | Down | Natural killer cell mediated cytotoxicity (mmu04650)  Protein processing in endoplasmic reticulum (mmu04141) | 1.81  1.64 |
|  | Cluster 4 | Down | ECM-receptor interaction (mmu04512)  Platelet activation (mmu04611)  Hematopoietic cell lineage (mmu04640)  Viral protein interaction with cytokine and cytokine receptor (mmu04061)  Cytokine-cytokine receptor interaction (mmu04060) | 2.16  2.02  2.02  1.86  1.53 |
|  | Cluster 5 | Up | N/A | N/A |
|  | Cluster 6 | Up | Circadian rhythm (mmu04710)  Circadian entrainment (mmu04713) | 2.57  2.05 |
|  | Cluster 7 | Up | Non-alcoholic fatty liver disease (mmu04932) | 2.0 |
| NI vs OB | Cluster 1 | Down | Fat digestion and absorption (mmu04975)  Pancreatic secretion (mmu04972)  Protein digestion and absorption (mmu04974)  Glycerolipid metabolism (mmu00561)  Influenza A (mmu05164) | 2.25  2.2  2.01  1.9  1.34 |
|  | Cluster 2 | Down | N/A | N/A |
| NL vs NI | Cluster 1 | Up | Legionellosis (mmu05134)  Longevity regulating pathway - multiple species (mmu04213)  Protein processing in endoplasmic reticulum (mmu04141)  Antigen processing and presentation (mmu04612)  Toxoplasmosis (mmu05145) | 2.09  2.08  2.04  2.0  1.84 |
|  | Cluster 2 | Up | N/A | N/A |

**Table S2** The function of important hub genes in UniProt database

| Groups | Hub genes | Description | Function |
| --- | --- | --- | --- |
| NI vs UN cluster 1 | *Camp* | Cathelicidin antimicrobial peptide | Binds to bacterial lipopolysaccharides (LPS) and has antibacterial activity |
|  | *Ctsg* | Cathepsin G | Serine protease with trypsin- and chymotrypsin-like specificity and also displays antibacterial activity |
|  | *Elane* | Neutrophil elastase | Medullasin modifies the functions of natural killer cells, monocytes and granulocytes |
|  | *Il1b* | Interleukin-1 beta | Potent pro-inflammatory cytokine |
|  | *Lcn2* | Neutrophil gelatinase-associated lipocalin | Iron-trafficking protein involved in multiple processes such as apoptosis, innate immunity and renal development |
|  | *Ltf* | Lactotransferrin | A major iron-binding and multifunctional protein found in exocrine fluids and has antimicrobial activity |
|  | *Ly6g* | Lymphocyte antigen 6G | Expressed in granulocyte and 59 other tissues |
|  | *Mpo* | Myeloperoxidase | Part of the host defense system of polymorphonuclear leukocytes |
|  | *Prtn3* | Myeloblastin | Serine protease that degrades elastin, fibronectin, laminin, vitronectin, and collagen types I, III, and IV |
|  | *S100a8* | Protein S100-A8 | A calcium- and zinc-binding protein which plays a prominent role in the regulation of inflammatory processes and immune response |
|  | *S100a9* | Protein S100-A9 | A calcium- and zinc-binding protein which plays a prominent role in the regulation of inflammatory processes and immune response |
| NI vs UN cluster 3 | *Dnaja1* | DnaJ homolog subfamily A member 1 | Co-chaperone for HSPA8/Hsc70 and co-chaperone for HSPA1B which protect cells against apoptosis |
|  | *Gzma* | Granzyme A | Abundant protease in the cytosolic granules of cytotoxic T-cells and NK-cells which activates caspase-independent pyroptosis when delivered into the target cell through the immunological synapse |
|  | *Gzmb* | Granzyme B | Abundant protease in the cytosolic granules of cytotoxic T-cells and NK-cells which activates caspase-independent pyroptosis when delivered into the target cell through the immunological synapse |
|  | *Hspa1a* | Heat shock 70 kDa protein 1A | Molecular chaperone implicated in a wide variety of cellular processes |
|  | *Hspa1b* | Heat shock 70 kDa protein 1B | Molecular chaperone implicated in a wide variety of cellular processes |
|  | *Hspb7* | Heat shock protein beta-7 | Molecular chaperone participates in stress response |
|  | *Hsph1* | Heat shock protein 105 kDa | Inhibits HSPA8/HSC70 ATPase and chaperone activities |
|  | *Klra1* | Killer cell lectin-like receptor subfamily A member 1 | Carbohydrate binding |
|  | *Klra7* | Killer cell lectin-like receptor 7 | Receptor on NK cells for class I MHC |
|  | *Klrg1* | Killer cell lectin-like receptor subfamily G member 1 | Plays an inhibitory role on natural killer (NK) cells and T-cell functions upon binding to their non-MHC ligands |
|  | *Ncr1* | Natural cytotoxicity triggering receptor 1 | Cytotoxicity-activating receptor that may contribute to the increased efficiency of activated NK cells to mediate tumor cell lysis |
|  | *Serpinb9b* | serine (or cysteine) peptidase inhibitor, clade B, member 9b | Cysteine-type endopeptidase inhibitor activity involved in apoptotic process, serine-type endopeptidase inhibitor activity and protease binding |
| NI vs UN cluster 4 | *Gp1ba* | Platelet glycoprotein Ib alpha chain | A surface membrane protein of platelets, participates in the formation of platelet plugs by binding to the A1 domain of vWF |
|  | *Gp5* | Platelet glycoprotein V | The GPIb-V-IX complex functions as the vWF receptor and mediates vWF-dependent platelet adhesion to blood vessels |
|  | *Gp6* | Platelet glycoprotein VI | Collagen receptor involved in collagen-induced platelet adhesion and activation |
|  | *Itga2b* | Integrin alpha-IIb | A receptor for fibronectin, fibrinogen, plasminogen, prothrombin, thrombospondin and vitronectin |
|  | *Mpl* | Thrombopoietin receptor | Receptor for thrombopoietin that acts as a primary regulator of megakaryopoiesis and platelet production |
|  | *Pf4* | Platelet factor 4 | Released during platelet aggregation, and chemotactic for neutrophils and monocytes |
|  | *Ppbp* | Pro-platelet basic protein | Participate in platelet degranulation and neutrophil degranulation |
| NI vs OB cluster 1 | *Cel* | Carboxyl ester lipase | Catalyzes the hydrolysis of a wide range of substrates including cholesteryl esters, phospholipids, lysophospholipids, di- and tri-acylglycerols, and fatty acid esters of hydroxy fatty acids |
|  | *Cela2a* | Chymotrypsin-like elastase family member 2A | Elastase that enhances insulin signaling and might have a physiologic role in cellular glucose metabolism |
|  | *Cela3b* | Chymotrypsin-like elastase family member 3B | Efficient protease with alanine specificity but only little elastolytic activity |
|  | *Clps* | Colipase | A co-factor of pancreatic lipase |
|  | *Cpa1* | Carboxypeptidase A1 | Carboxypeptidase that catalyzes the release of a C-terminal amino acid |
|  | *Cpa2* | Carboxypeptidase A2 | Similar to that of carboxypeptidase A (EC 3.4.17.1), but with a preference for bulkier C-terminal residues |
|  | *Cpb1* | Carboxypeptidase B1 | Participate in metabolism of angiotensinogen to angiotensins |
|  | *Ctrb1* | Chymotrypsinogen B | Activation of matrix metalloproteinases |
|  | *Pla2g1b* | Phospholipase A2 | May play a role in the biosynthesis of N-acyl ethanolamines that regulate energy metabolism and inflammation in the intestinal tract |
|  | *Pnlip* | Pancreatic triacylglycerol lipase | Plays an important role in fat metabolism |
|  | *Pnliprp1* | Inactive pancreatic lipase-related protein 1 | May function as inhibitor of dietary triglyceride digestion |
|  | *Pnliprp2* | Pancreatic lipase-related protein 2 | Lipase that primarily hydrolyzes triglycerides and galactosylglycerides |
|  | *Prss3* | Protease, serine 3 | Serine-type endopeptidase activity, serine-type peptidase activity and calcium ion binding |
|  | *Reg1* | Lithostathine-1 | Might act as an inhibitor of spontaneous calcium carbonate precipitation |
|  | *Reg2* | Lithostathine-2 | Might act as an inhibitor of spontaneous calcium carbonate precipitation |
|  | *Sycn* | Syncollin | Functions in exocytosis in pancreatic acinar cells regulating the fusion of zymogen granules with each other |
|  | *Try5* | Trypsin 5 | Serine-type endopeptidase activity, serine-type peptidase activity and calcium ion binding |
|  | *2210010C04Rik* | RIKEN cDNA 2210010C04 gene | Serine-type endopeptidase activity |
| NL vs NI  Cluster 1 | *Ahsa2* | Activator of 90 kDa heat shock protein ATPase homolog 2 | Co-chaperone that stimulates HSP90 ATPase activity |
|  | *Dnaja1* | DnaJ homolog subfamily A member 1 | Co-chaperone for HSPA8/Hsc70 and co-chaperone for HSPA1B which protect cells against apoptosis |
|  | *Dnajb1* | DnaJ homolog subfamily B member 1 | Interacts with HSP70 and can stimulate its ATPase activity and stimulates the association between HSC70 and HIP |
|  | *Hspa1a* | Heat shock 70 kDa protein 1A | Molecular chaperone implicated in a wide variety of cellular processes |
|  | *Hspa1b* | Heat shock 70 kDa protein 1B | Molecular chaperone implicated in a wide variety of cellular processes |
|  | *Hsph1* | Heat shock protein 105 kDa | Inhibits HSPA8/HSC70 ATPase and chaperone activities |
